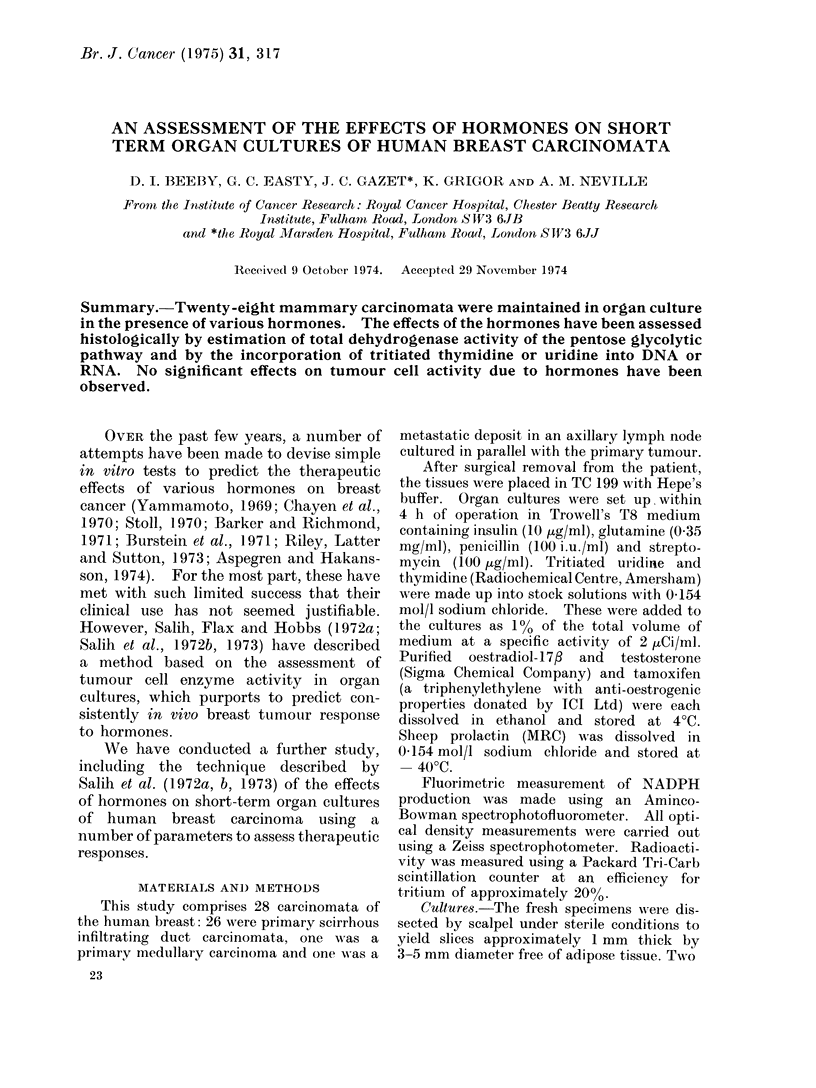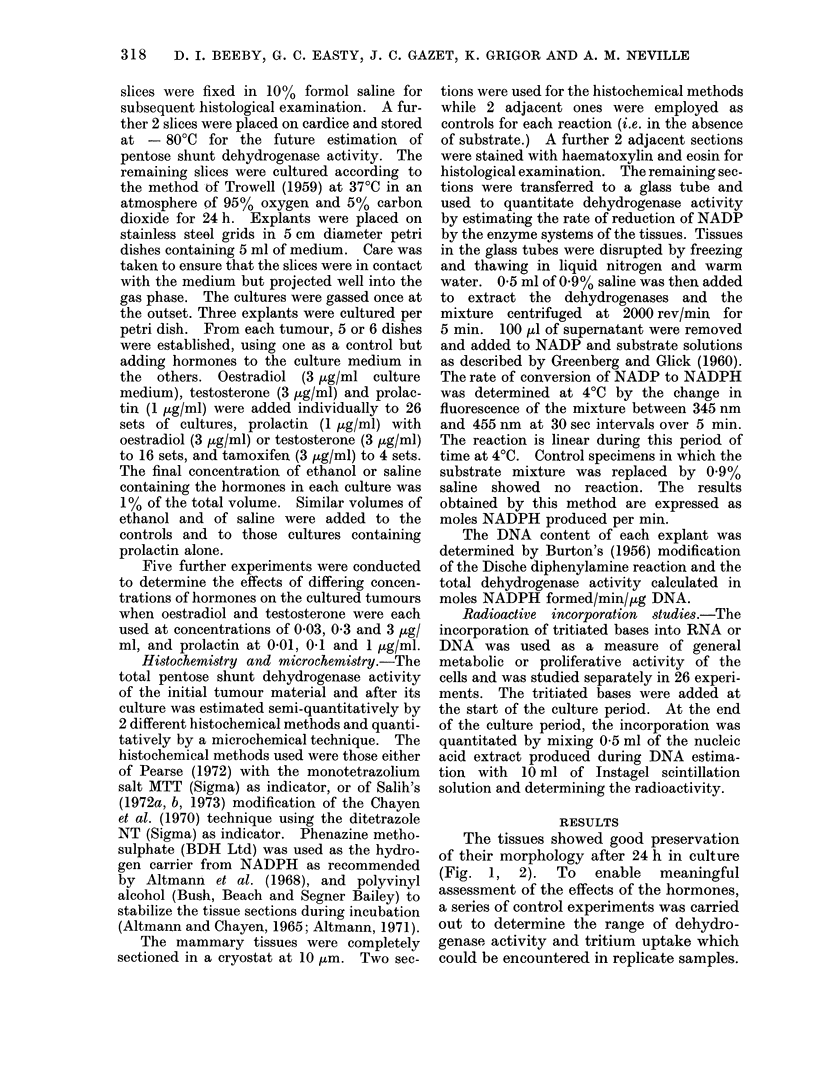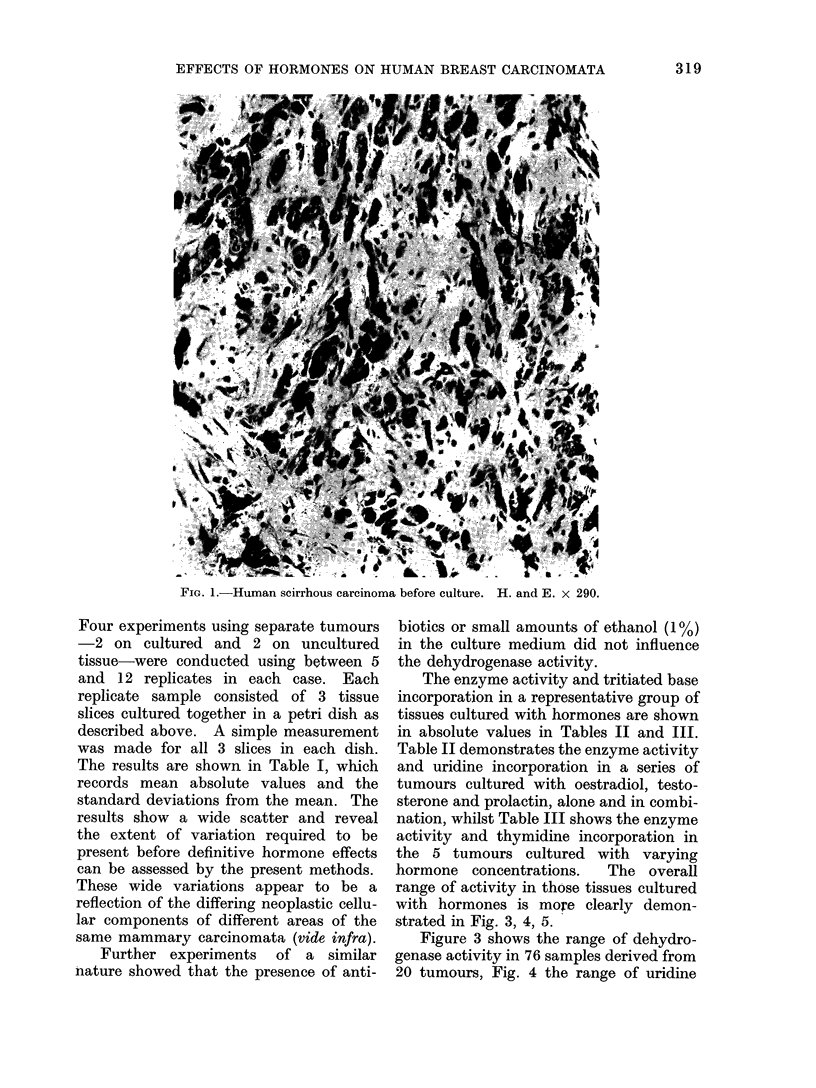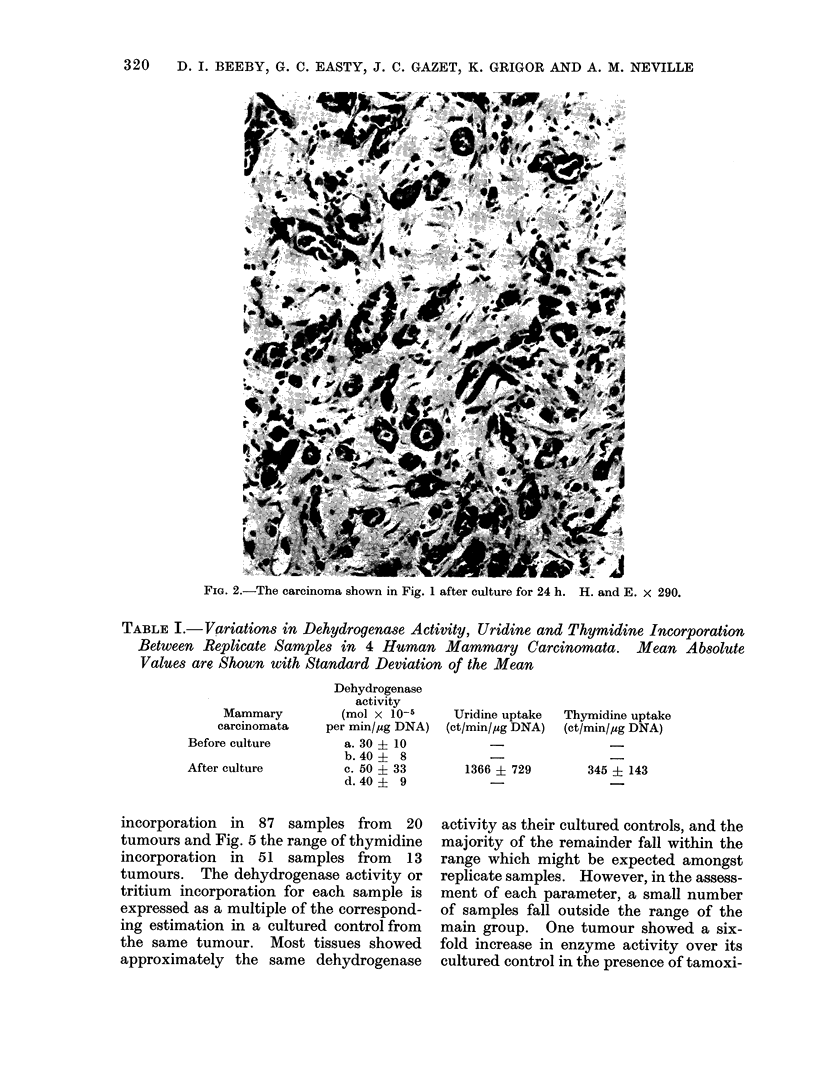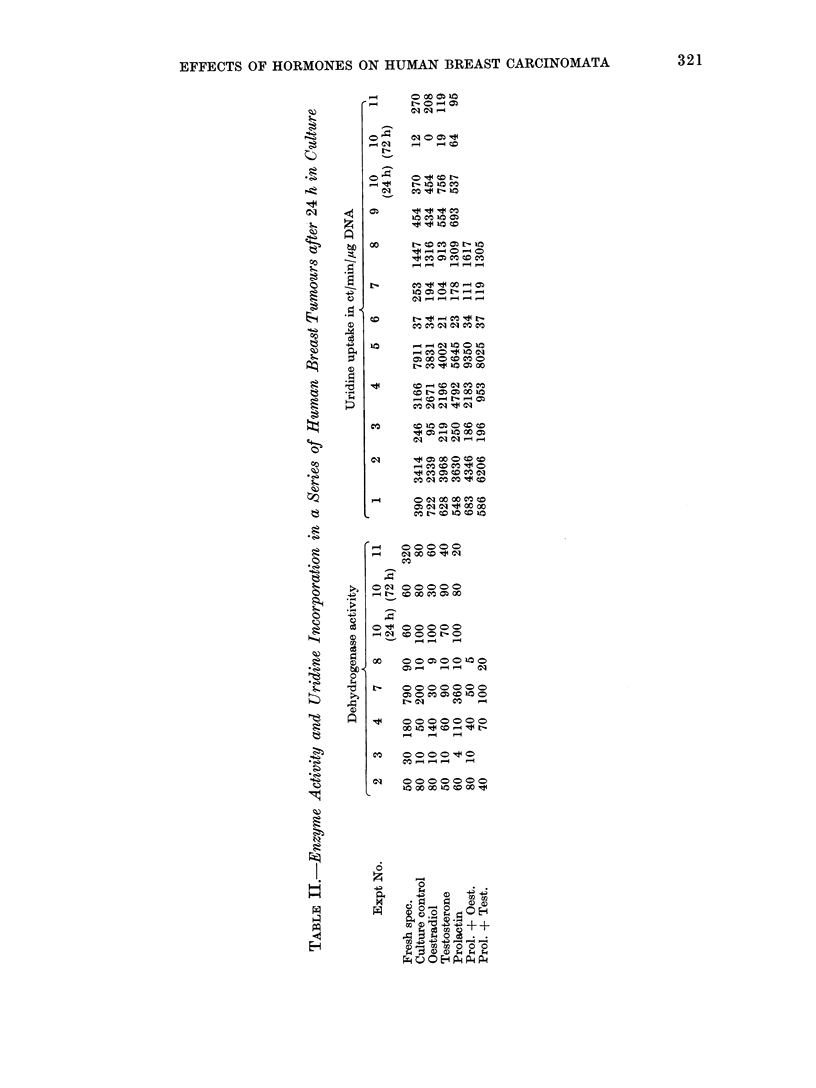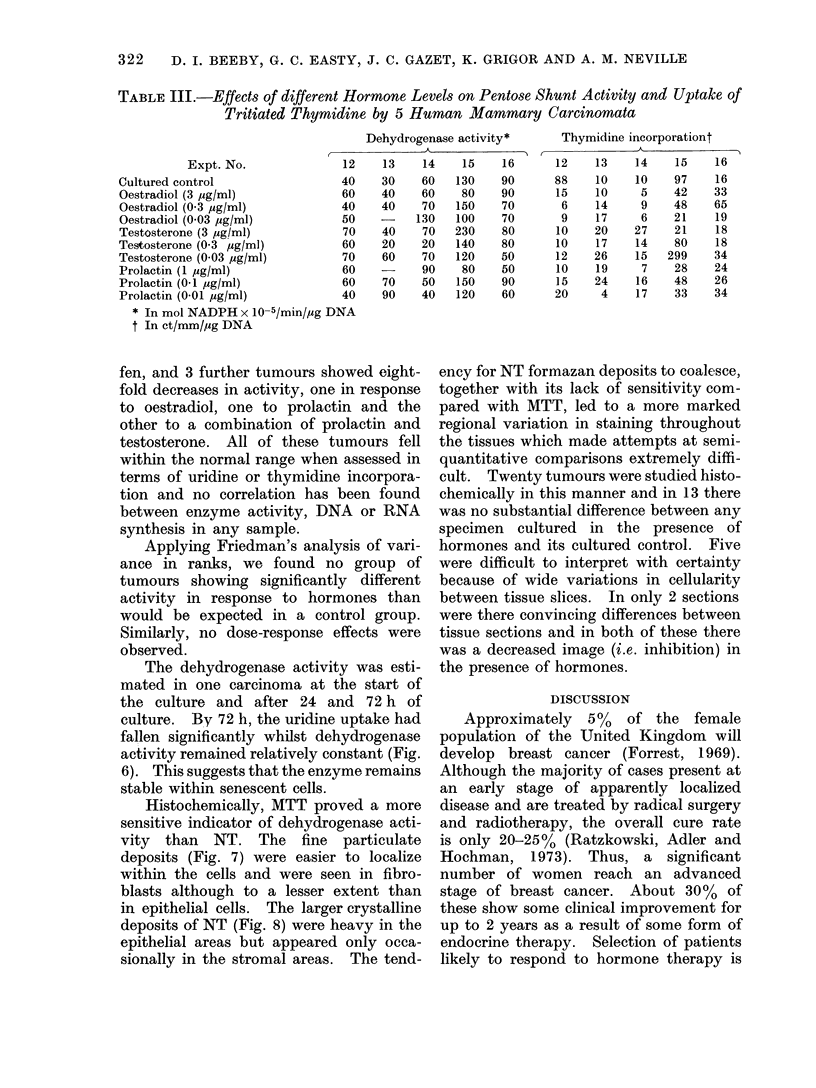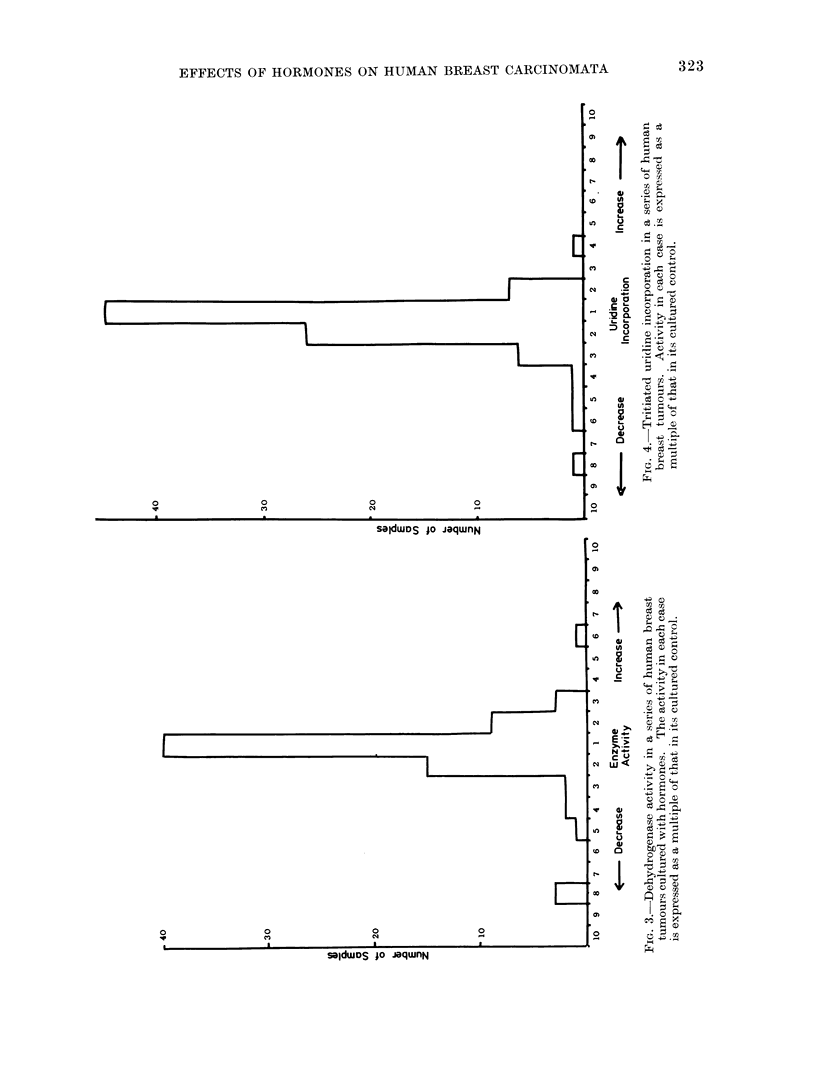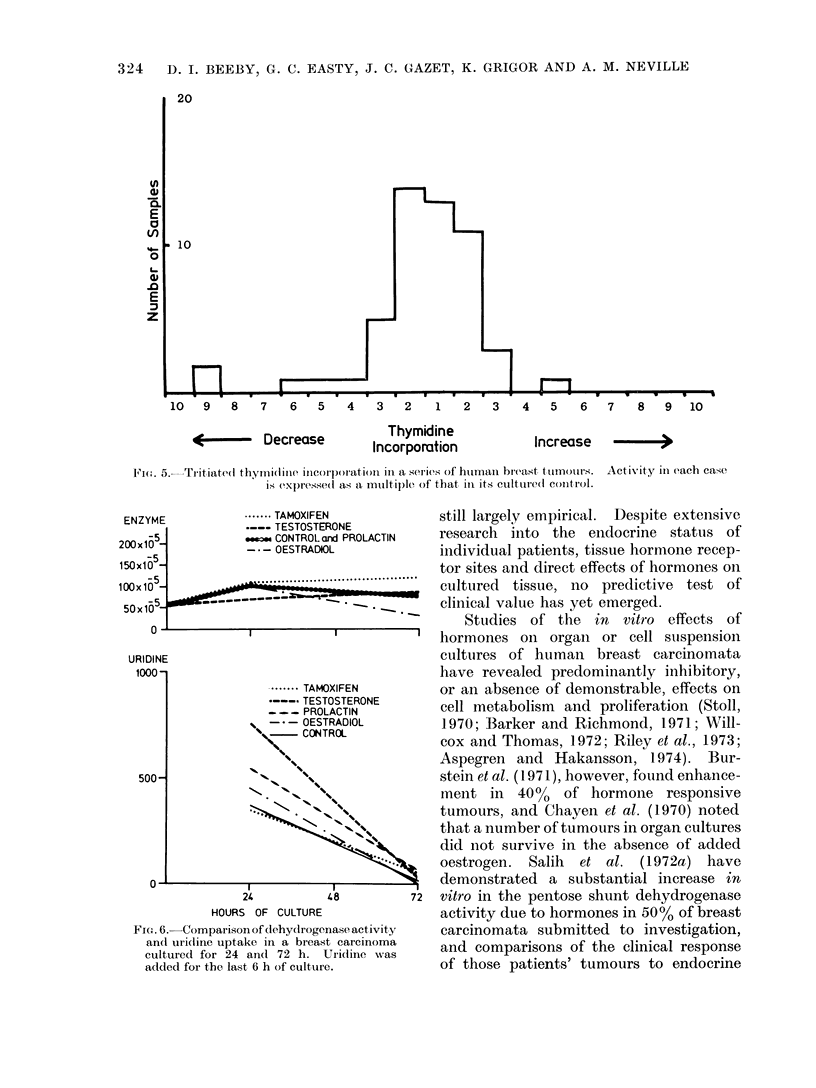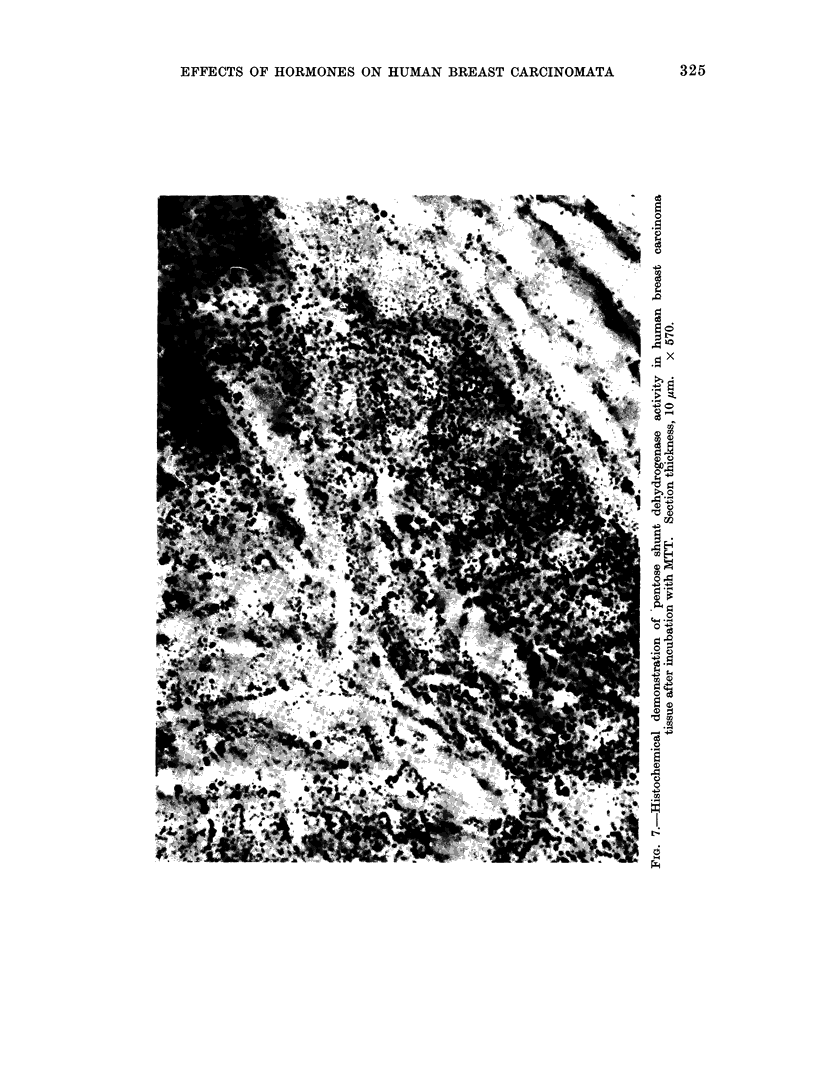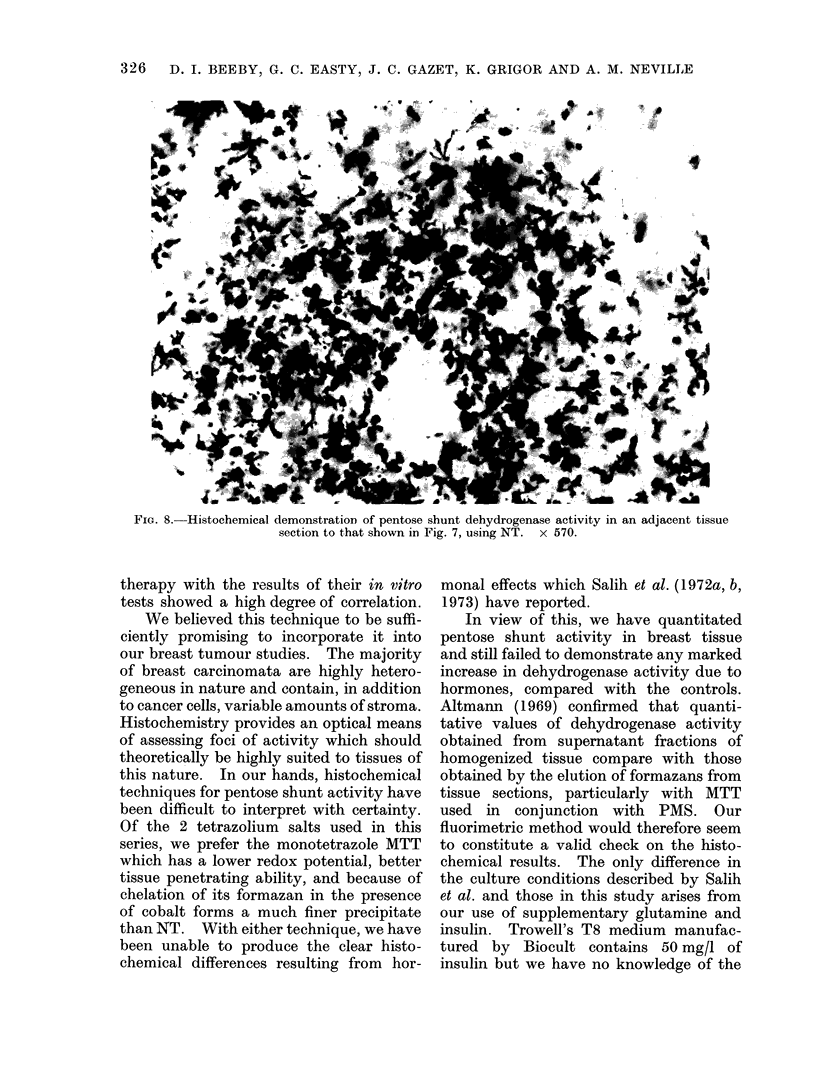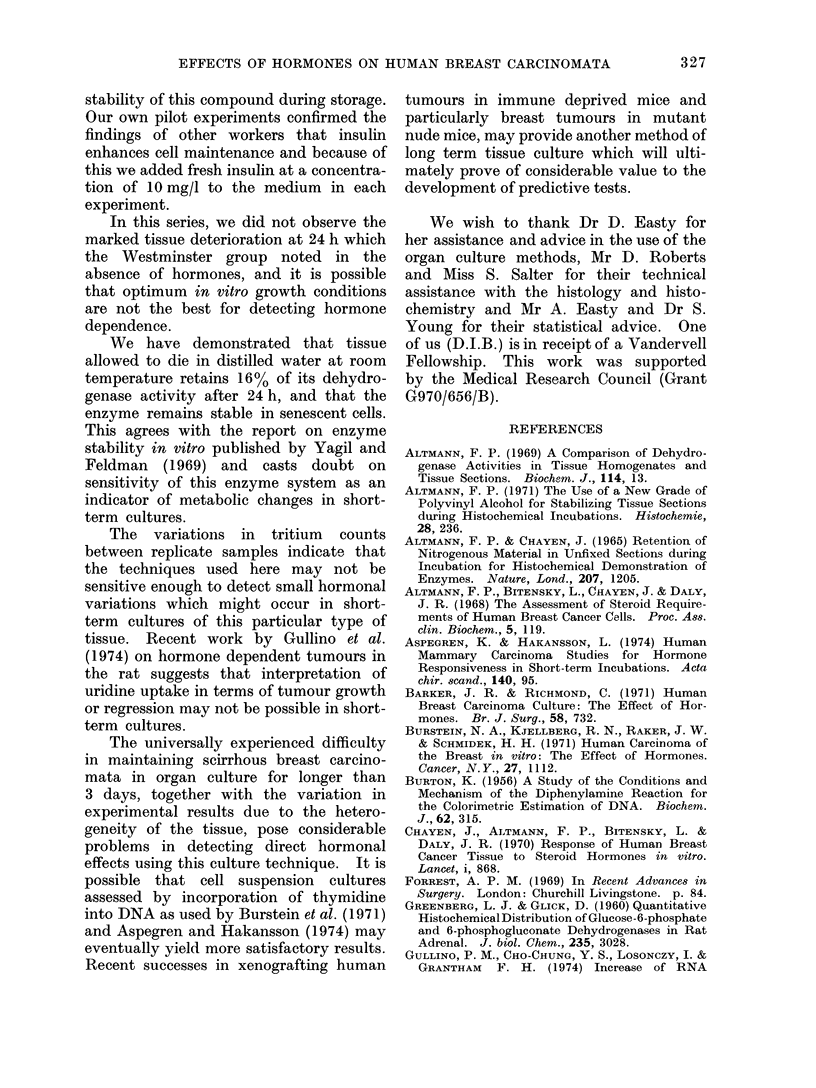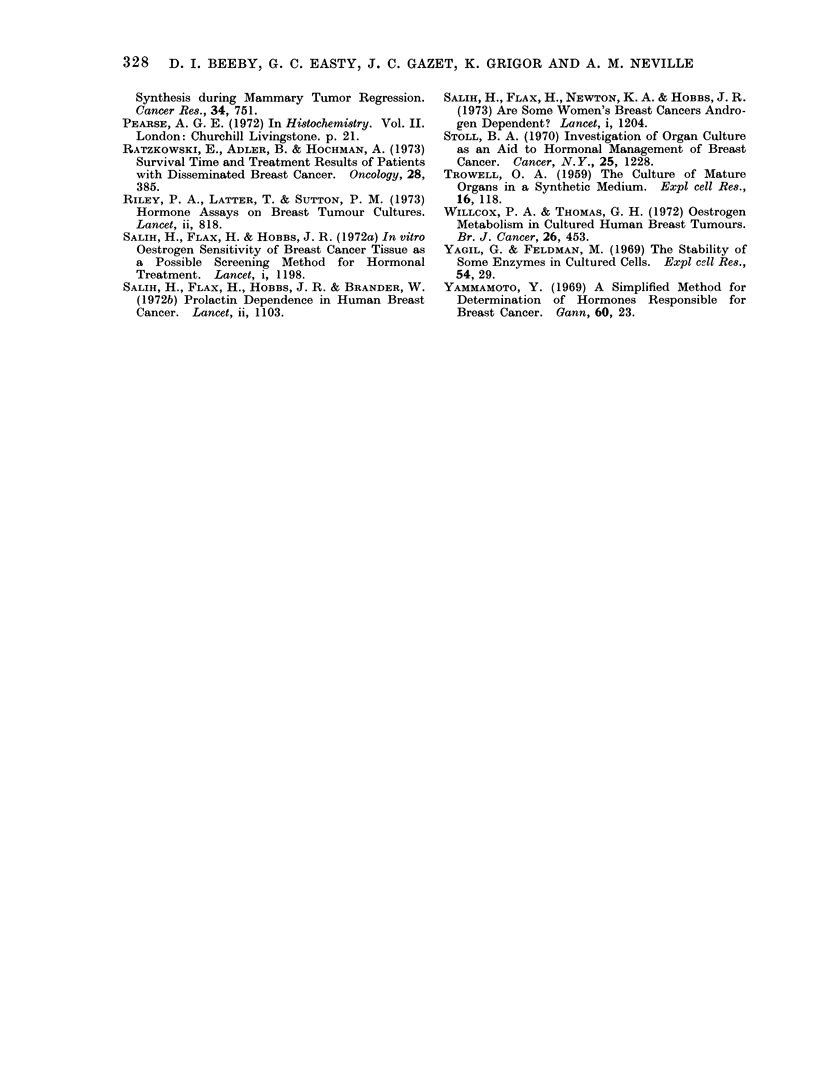# An assessment of the effects of hormones on short term organ cultures of human breast carcinomata.

**DOI:** 10.1038/bjc.1975.66

**Published:** 1975-03

**Authors:** D. I. Beeby, G. C. Easty, J. C. Gazet, K. Grigor, A. M. Neville

## Abstract

**Images:**


*Br. J. Cancer* (1975) **31**, 317

### AN ASSESSMENT OF THE EFFECTS OF HORMONES ON SHORT TERM ORGAN CULTURES OF HUMAN BREAST CARCINOMATA

D. I. BEEBY, G. C. EASTY, J. C. GAZET*, K. GRIGOR AND A. M. NEVILLE

*From the Institute of Cancer Research: Royal Cancer Hospital, Chester Beatty Research Institute, Fulham Road, London SW3 6JB and *the Royal Marsden Hospital, Fulham Road, London SW3 6JJ*

Received 9 October 1974. Accepted 29 November 1974

**Summary.—Twenty-eight mammary carcinomata were maintained in organ culture in the presence of various hormones. The effects of the hormones have been assessed histologically by estimation of total dehydrogenase activity of the pentose glycolytic pathway and by the incorporation of tritiated thymidine or uridine into DNA or RNA. No significant effects on tumour cell activity due to hormones have been observed.**

OVER the past few years, a number of attempts have been made to devise simple *in vitro* tests to predict the therapeutic effects of various hormones on breast cancer (Yammamoto, 1969; Chayen *et al.*, 1970; Stoll, 1970; Barker and Richmond, 1971; Burstein *et al.*, 1971; Riley, Latter and Sutton, 1973; Aspegren and Hakansson, 1974). For the most part, these have met with such limited success that their clinical use has not seemed justifiable. However, Salih, Flax and Hobbs (1972*a*; Salih *et al.*, 1972*b*, 1973) have described a method based on the assessment of tumour cell enzyme activity in organ cultures, which purports to predict consistently *in vivo* breast tumour response to hormones.

We have conducted a further study, including the technique described by Salih *et al.* (1972*a*, *b*, 1973) of the effects of hormones on short-term organ cultures of human breast carcinoma using a number of parameters to assess therapeutic responses.

### MATERIALS AND METHODS

This study comprises 28 carcinomata of the human breast: 26 were primary scirrhous infiltrating duct carcinomata, one was a primary medullary carcinoma and one was a metastatic deposit in an axillary lymph node cultured in parallel with the primary tumour.

After surgical removal from the patient, the tissues were placed in TC 199 with Hepe's buffer. Organ cultures were set up within 4 h of operation in Trowell's T8 medium containing insulin (10 μg/ml), glutamine (0·35 mg/ml), penicillin (100 i.u./ml) and streptomycin (100 μg/ml). Tritiated uridine and thymidine (Radiochemical Centre, Amersham) were made up into stock solutions with 0·154 mol/l sodium chloride. These were added to the cultures as 1% of the total volume of medium at a specific activity of 2 μCi/ml. Purified oestradiol-17β and testosterone (Sigma Chemical Company) and tamoxifen (a triphenylethylene with anti-oestrogenic properties donated by ICI Ltd) were each dissolved in ethanol and stored at 4°C. Sheep prolactin (MRC) was dissolved in 0·154 mol/l sodium chloride and stored at − 40°C.

Fluorimetric measurement of NADPH production was made using an Aminco-Bowman spectrophotofluorometer. All optical density measurements were carried out using a Zeiss spectrophotometer. Radioactivity was measured using a Packard Tri-Carb scintillation counter at an efficiency for tritium of approximately 20%.

*Cultures.*—The fresh specimens were dissected by scalpel under sterile conditions to yield slices approximately 1 mm thick by 3–5 mm diameter free of adipose tissue. Two slices were fixed in 10% formol saline for subsequent histological examination. A further 2 slices were placed on cardice and stored at $-80°C$ for the future estimation of pentose shunt dehydrogenase activity. The remaining slices were cultured according to the method of Trowell (1959) at 37°C in an atmosphere of 95% oxygen and 5% carbon dioxide for 24 h. Explants were placed on stainless steel grids in 5 cm diameter petri dishes containing 5 ml of medium. Care was taken to ensure that the slices were in contact with the medium but projected well into the gas phase. The cultures were gassed once at the outset. Three explants were cultured per petri dish. From each tumour, 5 or 6 dishes were established, using one as a control but adding hormones to the culture medium in the others. Oestradiol (3 $\mu$g/ml culture medium), testosterone (3 $\mu$g/ml) and prolactin (1 $\mu$g/ml) were added individually to 26 sets of cultures, prolactin (1 $\mu$g/ml) with oestradiol (3 $\mu$g/ml) or testosterone (3 $\mu$g/ml) to 16 sets, and tamoxifen (3 $\mu$g/ml) to 4 sets. The final concentration of ethanol or saline containing the hormones in each culture was 1% of the total volume. Similar volumes of ethanol and of saline were added to the controls and to those cultures containing prolactin alone.

Five further experiments were conducted to determine the effects of differing concentrations of hormones on the cultured tumours when oestradiol and testosterone were each used at concentrations of 0·03, 0·3 and 3 $\mu$g/ml, and prolactin at 0·01, 0·1 and 1 $\mu$g/ml.

*Histochemistry and microchemistry*.—The total pentose shunt dehydrogenase activity of the initial tumour material and after its culture was estimated semi-quantitatively by 2 different histochemical methods and quantitatively by a microchemical technique. The histochemical methods used were those either of Pearse (1972) with the monotetrazolium salt MTT (Sigma) as indicator, or of Salih's (1972*a*, *b*, 1973) modification of the Chayen *et al.* (1970) technique using the ditetrazole NT (Sigma) as indicator. Phenazine methosulphate (BDH Ltd) was used as the hydrogen carrier from NADPH as recommended by Altmann *et al.* (1968), and polyvinyl alcohol (Bush, Beach and Segner Bailey) to stabilize the tissue sections during incubation (Altmann and Chayen, 1965; Altmann, 1971).

The mammary tissues were completely sectioned in a cryostat at 10 $\mu$m. Two sections were used for the histochemical methods while 2 adjacent ones were employed as controls for each reaction (*i.e.* in the absence of substrate.) A further 2 adjacent sections were stained with haematoxylin and eosin for histological examination. The remaining sections were transferred to a glass tube and used to quantitate dehydrogenase activity by estimating the rate of reduction of NADP by the enzyme systems of the tissues. Tissues in the glass tubes were disrupted by freezing and thawing in liquid nitrogen and warm water. 0·5 ml of 0·9% saline was then added to extract the dehydrogenases and the mixture centrifuged at 2000 rev/min for 5 min. 100 $\mu$l of supernatant were removed and added to NADP and substrate solutions as described by Greenberg and Glick (1960). The rate of conversion of NADP to NADPH was determined at 4°C by the change in fluorescence of the mixture between 345 nm and 455 nm at 30 sec intervals over 5 min. The reaction is linear during this period of time at 4°C. Control specimens in which the substrate mixture was replaced by 0·9% saline showed no reaction. The results obtained by this method are expressed as moles NADPH produced per min.

The DNA content of each explant was determined by Burton's (1956) modification of the Dische diphenylamine reaction and the total dehydrogenase activity calculated in moles NADPH formed/min/$\mu$g DNA.

*Radioactive incorporation studies*.—The incorporation of tritiated bases into RNA or DNA was used as a measure of general metabolic or proliferative activity of the cells and was studied separately in 26 experiments. The tritiated bases were added at the start of the culture period. At the end of the culture period, the incorporation was quantitated by mixing 0·5 ml of the nucleic acid extract produced during DNA estimation with 10 ml of Instagel scintillation solution and determining the radioactivity.

### RESULTS

The tissues showed good preservation of their morphology after 24 h in culture (Fig. 1, 2). To enable meaningful assessment of the effects of the hormones, a series of control experiments was carried out to determine the range of dehydrogenase activity and tritium uptake which could be encountered in replicate samples.

FIG. 1.—Human scirrhous carcinoma before culture. H. and E. × 290.

Four experiments using separate tumours —2 on cultured and 2 on uncultured tissue—were conducted using between 5 and 12 replicates in each case. Each replicate sample consisted of 3 tissue slices cultured together in a petri dish as described above. A simple measurement was made for all 3 slices in each dish. The results are shown in Table I, which records mean absolute values and the standard deviations from the mean. The results show a wide scatter and reveal the extent of variation required to be present before definitive hormone effects can be assessed by the present methods. These wide variations appear to be a reflection of the differing neoplastic cellular components of different areas of the same mammary carcinomata (*vide infra*).

Further experiments of a similar nature showed that the presence of antibiotics or small amounts of ethanol (1%) in the culture medium did not influence the dehydrogenase activity.

The enzyme activity and tritiated base incorporation in a representative group of tissues cultured with hormones are shown in absolute values in Tables II and III. Table II demonstrates the enzyme activity and uridine incorporation in a series of tumours cultured with oestradiol, testosterone and prolactin, alone and in combination, whilst Table III shows the enzyme activity and thymidine incorporation in the 5 tumours cultured with varying hormone concentrations. The overall range of activity in those tissues cultured with hormones is more clearly demonstrated in Fig. 3, 4, 5.

Figure 3 shows the range of dehydrogenase activity in 76 samples derived from 20 tumours, Fig. 4 the range of uridine

FIG. 2.—The carcinoma shown in Fig. 1 after culture for 24 h. H. and E. × 290.

TABLE I.—*Variations in Dehydrogenase Activity, Uridine and Thymidine Incorporation Between Replicate Samples in 4 Human Mammary Carcinomata. Mean Absolute Values are Shown with Standard Deviation of the Mean*

| Mammary carcinomata | Dehydrogenase activity (mol × $10^{-5}$ per min/μg DNA) | Uridine uptake (ct/min/μg DNA) | Thymidine uptake (ct/min/μg DNA) |
|---|---|---|---|
| Before culture | a. 30 ± 10 | — | — |
| | b. 40 ± 8 | — | — |
| After culture | c. 50 ± 33 | 1366 ± 729 | 345 ± 143 |
| | d. 40 ± 9 | — | — |

incorporation in 87 samples from 20 tumours and Fig. 5 the range of thymidine incorporation in 51 samples from 13 tumours. The dehydrogenase activity or tritium incorporation for each sample is expressed as a multiple of the corresponding estimation in a cultured control from the same tumour. Most tissues showed approximately the same dehydrogenase activity as their cultured controls, and the majority of the remainder fall within the range which might be expected amongst replicate samples. However, in the assessment of each parameter, a small number of samples fall outside the range of the main group. One tumour showed a six-fold increase in enzyme activity over its cultured control in the presence of tamoxi-

TABLE II.—*Enzyme Activity and Uridine Incorporation in a Series of Human Breast Tumours after 24 h in Culture*

| | Dehydrogenase activity | | | | | | | | Uridine uptake in ct/min/μg DNA | | | | | | | | | | | |
|---|---|---|---|---|---|---|---|---|---|---|---|---|---|---|---|---|---|---|---|---|
| Expt No. | 2 | 3 | 4 | 7 | 8 | 10 (24 h) | 10 (72 h) | 11 | 1 | 2 | 3 | 4 | 5 | 6 | 7 | 8 | 9 | 10 (24 h) | 10 (72 h) | 11 |
| Fresh spec. | 50 | 30 | 180 | 790 | 90 | 60 | 60 | 320 | | | | | | | | | | | | |
| Culture control | 80 | 10 | 50 | 200 | 10 | 100 | 80 | 80 | 390 | 3414 | 246 | 3166 | 7911 | 37 | 253 | 1447 | 454 | 370 | 12 | 270 |
| Oestradiol | 80 | 10 | 140 | 30 | 9 | 100 | 30 | 60 | 722 | 2339 | 95 | 2671 | 3831 | 34 | 194 | 1316 | 434 | 454 | 0 | 208 |
| Testosterone | 50 | 10 | 60 | 90 | 10 | 70 | 90 | 40 | 628 | 3968 | 219 | 2196 | 4002 | 21 | 104 | 913 | 554 | 756 | 19 | 119 |
| Prolactin | 60 | 4 | 110 | 360 | 10 | 100 | 80 | 20 | 548 | 3630 | 250 | 4792 | 5645 | 23 | 178 | 1309 | 693 | 537 | 64 | 95 |
| Prol. + Oest. | 80 | 10 | 40 | 50 | 5 | | | | 683 | 4346 | 186 | 2183 | 9350 | 34 | 111 | 1617 | | | | |
| Prol. + Test. | 40 | | 70 | 100 | 20 | | | | 586 | 6206 | 196 | 953 | 8025 | 37 | 119 | 1305 | | | | |

TABLE III.—*Effects of different Hormone Levels on Pentose Shunt Activity and Uptake of Tritiated Thymidine by 5 Human Mammary Carcinomata*

| | Dehydrogenase activity* | | | | | Thymidine incorporation† | | | | |
|---|---|---|---|---|---|---|---|---|---|---|
| Expt. No. | 12 | 13 | 14 | 15 | 16 | 12 | 13 | 14 | 15 | 16 |
| Cultured control | 40 | 30 | 60 | 130 | 90 | 88 | 10 | 10 | 97 | 16 |
| Oestradiol (3 μg/ml) | 60 | 40 | 60 | 80 | 90 | 15 | 10 | 5 | 42 | 33 |
| Oestradiol (0·3 μg/ml) | 40 | 40 | 70 | 150 | 70 | 6 | 14 | 9 | 48 | 65 |
| Oestradiol (0·03 μg/ml) | 50 | — | 130 | 100 | 70 | 9 | 17 | 6 | 21 | 19 |
| Testosterone (3 μg/ml) | 70 | 40 | 70 | 230 | 80 | 10 | 20 | 27 | 21 | 18 |
| Testosterone (0·3 μg/ml) | 60 | 20 | 20 | 140 | 80 | 10 | 17 | 14 | 80 | 18 |
| Testosterone (0·03 μg/ml) | 70 | 60 | 70 | 120 | 50 | 12 | 26 | 15 | 299 | 34 |
| Prolactin (1 μg/ml) | 60 | — | 90 | 80 | 50 | 10 | 19 | 7 | 28 | 24 |
| Prolactin (0·1 μg/ml) | 60 | 70 | 50 | 150 | 90 | 15 | 24 | 16 | 48 | 26 |
| Prolactin (0·01 μg/ml) | 40 | 90 | 40 | 120 | 60 | 20 | 4 | 17 | 33 | 34 |

* In mol NADPH $\times 10^{-5}$/min/μg DNA

† In ct/mm/μg DNA

fen, and 3 further tumours showed eightfold decreases in activity, one in response to oestradiol, one to prolactin and the other to a combination of prolactin and testosterone. All of these tumours fell within the normal range when assessed in terms of uridine or thymidine incorporation and no correlation has been found between enzyme activity, DNA or RNA synthesis in any sample.

Applying Friedman's analysis of variance in ranks, we found no group of tumours showing significantly different activity in response to hormones than would be expected in a control group. Similarly, no dose-response effects were observed.

The dehydrogenase activity was estimated in one carcinoma at the start of the culture and after 24 and 72 h of culture. By 72 h, the uridine uptake had fallen significantly whilst dehydrogenase activity remained relatively constant (Fig. 6). This suggests that the enzyme remains stable within senescent cells.

Histochemically, MTT proved a more sensitive indicator of dehydrogenase activity than NT. The fine particulate deposits (Fig. 7) were easier to localize within the cells and were seen in fibroblasts although to a lesser extent than in epithelial cells. The larger crystalline deposits of NT (Fig. 8) were heavy in the epithelial areas but appeared only occasionally in the stromal areas. The tendency for NT formazan deposits to coalesce, together with its lack of sensitivity compared with MTT, led to a more marked regional variation in staining throughout the tissues which made attempts at semiquantitative comparisons extremely difficult. Twenty tumours were studied histochemically in this manner and in 13 there was no substantial difference between any specimen cultured in the presence of hormones and its cultured control. Five were difficult to interpret with certainty because of wide variations in cellularity between tissue slices. In only 2 sections were there convincing differences between tissue sections and in both of these there was a decreased image (*i.e.* inhibition) in the presence of hormones.

### DISCUSSION

Approximately 5% of the female population of the United Kingdom will develop breast cancer (Forrest, 1969). Although the majority of cases present at an early stage of apparently localized disease and are treated by radical surgery and radiotherapy, the overall cure rate is only 20–25% (Ratzkowski, Adler and Hochman, 1973). Thus, a significant number of women reach an advanced stage of breast cancer. About 30% of these show some clinical improvement for up to 2 years as a result of some form of endocrine therapy. Selection of patients likely to respond to hormone therapy is

FIG. 3.—Dehydrogenase activity in a series of human breast tumours cultured with hormones. The activity in each case is expressed as a multiple of that in its cultured control.

FIG. 4.—Tritiated uridine incorporation in a series of human breast tumours. Activity in each case is expressed as a multiple of that in its cultured control.

FIG. 5.—Tritiated thymidine incorporation in a series of human breast tumours. Activity in each case is expressed as a multiple of that in its cultured control.

FIG. 6.—Comparison of dehydrogenase activity and uridine uptake in a breast carcinoma cultured for 24 and 72 h. Uridine was added for the last 6 h of culture.

still largely empirical. Despite extensive research into the endocrine status of individual patients, tissue hormone receptor sites and direct effects of hormones on cultured tissue, no predictive test of clinical value has yet emerged.

Studies of the *in vitro* effects of hormones on organ or cell suspension cultures of human breast carcinomata have revealed predominantly inhibitory, or an absence of demonstrable, effects on cell metabolism and proliferation (Stoll, 1970; Barker and Richmond, 1971; Willcox and Thomas, 1972; Riley *et al.*, 1973; Aspegren and Hakansson, 1974). Burstein *et al.* (1971), however, found enhancement in 40% of hormone responsive tumours, and Chayen *et al.* (1970) noted that a number of tumours in organ cultures did not survive in the absence of added oestrogen. Salih *et al.* (1972*a*) have demonstrated a substantial increase *in vitro* in the pentose shunt dehydrogenase activity due to hormones in 50% of breast carcinomata submitted to investigation, and comparisons of the clinical response of those patients' tumours to endocrine

Fig. 7.—Histochemical demonstration of pentose shunt dehydrogenase activity in human breast carcinoma tissue after incubation with MTT. Section thickness, 10 μm. × 570.

FIG. 8.—Histochemical demonstration of pentose shunt dehydrogenase activity in an adjacent tissue section to that shown in Fig. 7, using NT. × 570.

therapy with the results of their *in vitro* tests showed a high degree of correlation.

We believed this technique to be sufficiently promising to incorporate it into our breast tumour studies. The majority of breast carcinomata are highly heterogeneous in nature and contain, in addition to cancer cells, variable amounts of stroma. Histochemistry provides an optical means of assessing foci of activity which should theoretically be highly suited to tissues of this nature. In our hands, histochemical techniques for pentose shunt activity have been difficult to interpret with certainty. Of the 2 tetrazolium salts used in this series, we prefer the monotetrazole MTT which has a lower redox potential, better tissue penetrating ability, and because of chelation of its formazan in the presence of cobalt forms a much finer precipitate than NT. With either technique, we have been unable to produce the clear histochemical differences resulting from hormonal effects which Salih *et al.* (1972*a*, *b*, 1973) have reported.

In view of this, we have quantitated pentose shunt activity in breast tissue and still failed to demonstrate any marked increase in dehydrogenase activity due to hormones, compared with the controls. Altmann (1969) confirmed that quantitative values of dehydrogenase activity obtained from supernatant fractions of homogenized tissue compare with those obtained by the elution of formazans from tissue sections, particularly with MTT used in conjunction with PMS. Our fluorimetric method would therefore seem to constitute a valid check on the histochemical results. The only difference in the culture conditions described by Salih *et al.* and those in this study arises from our use of supplementary glutamine and insulin. Trowell's T8 medium manufactured by Biocult contains 50 mg/l of insulin but we have no knowledge of the stability of this compound during storage. Our own pilot experiments confirmed the findings of other workers that insulin enhances cell maintenance and because of this we added fresh insulin at a concentration of 10 mg/l to the medium in each experiment.

In this series, we did not observe the marked tissue deterioration at 24 h which the Westminster group noted in the absence of hormones, and it is possible that optimum *in vitro* growth conditions are not the best for detecting hormone dependence.

We have demonstrated that tissue allowed to die in distilled water at room temperature retains 16% of its dehydrogenase activity after 24 h, and that the enzyme remains stable in senescent cells. This agrees with the report on enzyme stability *in vitro* published by Yagil and Feldman (1969) and casts doubt on sensitivity of this enzyme system as an indicator of metabolic changes in short-term cultures.

The variations in tritium counts between replicate samples indicate that the techniques used here may not be sensitive enough to detect small hormonal variations which might occur in short-term cultures of this particular type of tissue. Recent work by Gullino *et al.* (1974) on hormone dependent tumours in the rat suggests that interpretation of uridine uptake in terms of tumour growth or regression may not be possible in short-term cultures.

The universally experienced difficulty in maintaining scirrhous breast carcinomata in organ culture for longer than 3 days, together with the variation in experimental results due to the heterogeneity of the tissue, pose considerable problems in detecting direct hormonal effects using this culture technique. It is possible that cell suspension cultures assessed by incorporation of thymidine into DNA as used by Burstein *et al.* (1971) and Aspegren and Hakansson (1974) may eventually yield more satisfactory results. Recent successes in xenografting human tumours in immune deprived mice and particularly breast tumours in mutant nude mice, may provide another method of long term tissue culture which will ultimately prove of considerable value to the development of predictive tests.

We wish to thank Dr D. Easty for her assistance and advice in the use of the organ culture methods, Mr D. Roberts and Miss S. Salter for their technical assistance with the histology and histochemistry and Mr A. Easty and Dr S. Young for their statistical advice. One of us (D.I.B.) is in receipt of a Vandervell Fellowship. This work was supported by the Medical Research Council (Grant G970/656/B).

### REFERENCES

ALTMANN, F. P. (1969) A Comparison of Dehydrogenase Activities in Tissue Homogenates and Tissue Sections. *Biochem. J.*, **114**, 13.

ALTMANN, F. P. (1971) The Use of a New Grade of Polyvinyl Alcohol for Stabilizing Tissue Sections during Histochemical Incubations. *Histochemie*, **28**, 236.

ALTMANN, F. P. & CHAYEN, J. (1965) Retention of Nitrogenous Material in Unfixed Sections during Incubation for Histochemical Demonstration of Enzymes. *Nature, Lond.*, **207**, 1205.

ALTMANN, F. P., BITENSKY, L., CHAYEN, J. & DALY, J. R. (1968) The Assessment of Steroid Requirements of Human Breast Cancer Cells. *Proc. Ass. clin. Biochem.*, **5**, 119.

ASPEGREN, K. & HAKANSSON, L. (1974) Human Mammary Carcinoma Studies for Hormone Responsiveness in Short-term Incubations. *Acta chir. scand.*, **140**, 95.

BARKER, J. R. & RICHMOND, C. (1971) Human Breast Carcinoma Culture: The Effect of Hormones. *Br. J. Surg.*, **58**, 732.

BURSTEIN, N. A., KJELLBERG, R. N., RAKER, J. W. & SCHMIDEK, H. H. (1971) Human Carcinoma of the Breast *in vitro*: The Effect of Hormones. *Cancer, N.Y.*, **27**, 1112.

BURTON, K. (1956) A Study of the Conditions and Mechanism of the Diphenylamine Reaction for the Colorimetric Estimation of DNA. *Biochem. J.*, **62**, 315.

CHAYEN, J., ALTMANN, F. P., BITENSKY, L. & DALY, J. R. (1970) Response of Human Breast Cancer Tissue to Steroid Hormones *in vitro*. *Lancet*, i, 868.

FORREST, A. P. M. (1969) In *Recent Advances in Surgery*. London: Churchill Livingstone. p. 84.

GREENBERG, L. J. & GLICK, D. (1960) Quantitative Histochemical Distribution of Glucose-6-phosphate and 6-phosphogluconate Dehydrogenases in Rat Adrenal. *J. biol. Chem.*, **235**, 3028.

GULLINO, P. M., CHO-CHUNG, Y. S., LOSONCZY, I. & GRANTHAM F. H. (1974) Increase of RNA

Synthesis during Mammary Tumor Regression. *Cancer Res.*, **34**, 751.

PEARSE, A. G. E. (1972) In *Histochemistry*. Vol. II. London: Churchill Livingstone. p. 21.

RATZKOWSKI, E., ADLER, B. & HOCHMAN, A. (1973) Survival Time and Treatment Results of Patients with Disseminated Breast Cancer. *Oncology*, **28**, 385.

RILEY, P. A., LATTER, T. & SUTTON, P. M. (1973) Hormone Assays on Breast Tumour Cultures. *Lancet*, ii, 818.

SALIH, H., FLAX, H. & HOBBS, J. R. (1972*a*) *In vitro* Oestrogen Sensitivity of Breast Cancer Tissue as a Possible Screening Method for Hormonal Treatment. *Lancet*, i, 1198.

SALIH, H., FLAX, H., HOBBS, J. R. & BRANDER, W. (1972*b*) Prolactin Dependence in Human Breast Cancer. *Lancet*, ii, 1103.

SALIH, H., FLAX, H., NEWTON, K. A. & HOBBS, J. R. (1973) Are Some Women's Breast Cancers Androgen Dependent? *Lancet*, i, 1204.

STOLL, B. A. (1970) Investigation of Organ Culture as an Aid to Hormonal Management of Breast Cancer. *Cancer, N.Y.*, **25**, 1228.

TROWELL, O. A. (1959) The Culture of Mature Organs in a Synthetic Medium. *Expl cell Res.*, **16**, 118.

WILLCOX, P. A. & THOMAS, G. H. (1972) Oestrogen Metabolism in Cultured Human Breast Tumours. *Br. J. Cancer*, **26**, 453.

YAGIL, G. & FELDMAN, M. (1969) The Stability of Some Enzymes in Cultured Cells. *Expl cell Res.*, **54**, 29.

YAMMAMOTO, Y. (1969) A Simplified Method for Determination of Hormones Responsible for Breast Cancer. *Gann*, **60**, 23.